# Efficacy of *SGPP2* Modulation-Mediated Materials in Ameliorating Facial Wrinkles and Pore Sagging

**DOI:** 10.3390/cimb46080539

**Published:** 2024-08-20

**Authors:** Juhyun Kim, Sanghyun Ye, Seung-Hyun Jun, Nae-Gyu Kang

**Affiliations:** LG Household & Health Care (LG H&H) R&D Center, Seoul 07795, Republic of Korea; juhyunkim@lghnh.com (J.K.); shye123@lghnh.com (S.Y.)

**Keywords:** skin aging, skin wrinkle, wrinkle improvement, pore sagging, inflammation, cosmetics

## Abstract

Skin aging is a complex process with internal and external factors. Recent studies have suggested that enlargement and elongation of skin pores may be early signs of aging in addition to wrinkles and loss of elasticity. This study explores the potential of targeting the *SGPP2* gene in keratinocytes to address these emerging concerns. Using siRNA knockdown, we demonstrated that *SGPP2* modulates the production of inflammatory cytokines (interleukin (IL)-1β and IL-8). Furthermore, conditioned media experiments revealed that keratinocytes with high *SGPP2* expression indirectly influence fibroblast extracellular matrix remodeling, potentially contributing to enlarged pores and wrinkle formation. Based on these findings, we explored a complex formulation containing four *SGPP2*-modulating compounds. In vitro and in vivo experiments demonstrated the efficacy of the formulation in mitigating fine wrinkles and pore enlargement. This study highlights the significant implications of developing a more effective antiaging cosmetic formulation by targeting underlying inflammatory processes that drive skin aging.

## 1. Introduction

Skin aging is a multi-factorial process driven by the intricate interplay of intrinsic (time, genetic factors, and hormonal changes) and extrinsic (ultraviolet (UV) exposure and pollution) factors. Among these factors, inflammation is pivotal in intrinsic aging, characterized by the progressive increase in inflammatory cytokines such as prostaglandin E2 (PGE-2), IL-1β, and tumor necrosis factor (TNF)-α [1,2]. A key regulator of this chronic inflammatory state, also known as senescent-associated secretory phenotype (SASP), is the nuclear factor kappa-light-chain-enhancer of activated B cells (NF-κB) [3]. NF-κB upregulates various inflammatory cytokines and chemokines, including IL-6 and IL-8, as well as proteases such as matrix metalloproteinases (MMPs). This inflammatory milieu paracrinely induces senescence in neighboring cells, rendering them hypersensitive to external stimuli and primed for exaggerated inflammatory responses mediated by the p38 mitogen-activated protein kinase (MAPK) and NF-κB pathways [4]. Consequently, SASP factors like MMPs degrade surrounding extracellular matrix components, such as collagen and elastin, contributing to skin aging and wrinkle formation [5,6,7,8,9]. 

The clinical hallmarks of skin aging extend beyond wrinkle formation and encompass a range of manifestations, including reduced skin elasticity, epidermal thinning, and enlarged pores. Notably, enlarged pores are a cosmetic concern that involves not merely an uneven skin surface but is increasingly recognized as a precursor to wrinkle development [10]. With age, pores lengthen, expand, and coalesce, forming pre-wrinkles and exacerbating wrinkle severity [11,12,13]. 

Pore enlargement is associated with a complex interplay of factors, including skin elasticity, sebum level, and hormonal balance [14]. Elevated sebum production and reduced skin elasticity have been linked to wider pores. Beyond the enlargement, pore elongation is also a significant hallmark of skin aging. This elongation is attributed to a combination of factors, including the breakdown of dermal collagen and elastin fibers, inflammation, and alterations in the pore microenvironment [15,16,17]. While PGE-2 has been extensively studied in relation to pore enlargement [18], the roles of other inflammatory mediators and cytokines in this process remain largely unexplored. Consequently, clinical reports of pore enlargement or elongation are common; however, the underlying causes and effective treatment strategies remain poorly understood.

Recent studies have used genome-wide association studies (GWAS) to identify genetic markers associated with facial wrinkles [19,20], and some of these markers have been subjected to functional studies and gene regulation to determine whether they improve wrinkles [21,22]. Among the GWAS-identified genes implicated in wrinkle formation, we aimed to investigate the relationship between sphingosine 1-phosphate (S1P) phosphatase 2 (*SGPP2*), the gene encoding SGPP2 involved in inflammatory signaling [23], and the enlargement of pores, including elongation and wrinkle formation. SGPP2, an enzyme involved in the sphingolipid metabolic pathway, exhibits differential functions compared with its closely related paralog SGPP1 [24]. SGPP1 is involved in keratinocyte differentiation and skin homeostasis [25], whereas the role of SGPP2 in the skin is relatively not well understood. Given that S1P is downregulated in skin inflammatory conditions, such as atopic dermatitis, SGPP2 is upregulated in psoriasis, and SGPP2 is involved in inflammatory signaling in immune and endothelial cells [26,27], we hypothesized that SGPP2 contributes to inflammatory pathways in the skin. Notably, as an NF-κB-dependent gene that regulates pro-inflammatory cytokines IL-1β and IL-8, SGPP2 may play a role in skin cell aging and wrinkle formation related to SASP.

To elucidate the role of *SGPP2* in skin aging and wrinkle formation, we examined its expression and function in keratinocytes under inflammatory conditions. Using siRNA-knockdown, the study demonstrated that *SGPP2* modulates the production of inflammatory cytokines IL-1β and IL-8 in keratinocytes. Furthermore, conditioned media experiments revealed that keratinocytes with elevated *SGPP2* expression paracrinely modulate fibroblast extracellular matrix (ECM) remodeling, contributing to pore sagging and wrinkle formation.

Building upon these findings, the study explored the efficacy of four *SGPP2*-modulating compounds (fucoidan, feruloyl serotonin, hyaluronic acid, and *Polygonum cuspidatum* root extract) in a complex formulation. In vitro and in vivo experiments demonstrated the ability of this formulation to mitigate fine wrinkles and pore enlargement.

This study highlights the potential of *SGPP2* gene modulation as a therapeutic strategy to combat skin aging, particularly facial wrinkles, by targeting the underlying inflammatory processes. By regulating *SGPP2* expression and activity, effective control of inflammatory mediators is expected, alleviating skin aging and promoting skin health.

## 2. Materials and Methods

### 2.1. Cell Culture and Preparation

Human keratinocyte HaCaT cells (AddexBio, San Diego, CA, USA) were cultured in Dulbecco’s modified eagle medium (DMEM; Gibco, Grand Island, NY, USA) supplemented with 10% fetal bovine serum (FBS; Gibco), penicillin-streptomycin (Gibco), 1 mM sodium pyruvate (Gibco), 2 mM L-glutamine (Gibco), and 0.01 mM CaCl_2_ (Sigma-Aldrich, St. Louis, MO, USA) at 37 °C in a humidified atmosphere containing 5% CO_2_. Normal human dermal fibroblasts (NHDFs; ATCC, Manassas, VA, USA) were cultured in DMEM supplemented with 10% FBS and penicillin-streptomycin at 37 °C.

Hyaluronic acid, retinol, L-hydroxyproline, ginsenoside Rh1, cedrol, asiaticoside, luteolin, and D-panthenol were purchased from Sigma-Aldrich. Ronacare Balmance^®^ containing feruloyl serotonin and Fuligo^®^ containing fucoidan were purchased from Merck KGaA (Darmstadt, Germany) and Biospectrum, Inc. (Yongin, Republic of Korea), respectively. *Myristica fragrans* extract and *Polygonum cuspidatum* root extract were purchased from Bilco (Gunpo, Republic of Korea) and Alphacryptec (Cheongwon, Republic of Korea), respectively. *Polygonum cuspidatum* root extract, extracted with a water-butylene glycol mixture using a modified experimental method of Jeong et al. (2010) [28], is standardized to contain 4–6% polydatin as its active substance. *Myristica frangrans* extract, an ethanol-based extract, is standardized to contain a macelignan content of 2% or more. All extracts are managed in accordance with the manufacturer’s standards.

### 2.2. Transfection of Keratinocytes with siRNA for RNAi Experiments

Three types of *SGPP2* siRNAs and one negative control siRNA (siNC) were purchased from Bioneer (Daejeon, Republic of Korea), including AccuTarget™ genome-wide predesigned siRNA (SDH-1001) No. 130367-1, 130367-2, and 130367-3 for *SGPP2* and AccuTarget™ negative control siRNA (SN-1003). The siRNAs were dissolved in diethyl pyrocarbonate (DEPC)-treated distilled water to yield a final concentration of 100 μM. HaCaT cells were seeded at 2 × 10^5^ cells per well in a six-well plate and transfected with 100 nM of the siRNAs using Lipofectamine^®^ 2000 (Thermo Fisher Scientific, Waltham, MA, USA) following the manufacturer’s instructions. After transfection for 6 h, the cells were washed with phosphate-buffered saline. Subsequently, fresh DMEM supplemented with 10% FBS was added to each well. After incubation for 16 h, the cells were harvested for further analysis. The effectiveness of *SGPP2* knockdown was assessed using real-time quantitative polymerase chain reaction (RT-qPCR) and compared with that of cells treated with the negative control siRNA (siNC).

### 2.3. RNA Extraction and RT-qPCR

HaCaT and NHDFs were seeded in six-well plates at a density of 2 × 10^5^ and 3 × 10^5^ cells/well, respectively. After incubation for 24 h at 37 °C with 5% CO_2_, the cells were treated with different concentrations of the active ingredients for another 24 h in a serum-free medium. Following incubation, RNA was extracted from HaCaT and NHDFs using the RNeasy mini kit (Qiagen, Hilden, Germany). The concentration and purity of the extracted RNA were evaluated using a Nanodrop (Thermofisher, Wilmington, DE, USA). For further analysis, 1 μg of total RNA was used to generate cDNA through reverse transcription with a cDNA synthesis kit (Philekorea, Seoul, Republic of Korea), following the manufacturer’s protocol, and RT-qPCR was performed using the StepOnePlus^®^ Real-Time PCR System (Applied Biosystems, Waltham, MA, USA). The following TaqMan probes were used for RT-qPCR: *SGPP2* (Hs00544786_m1), collagen type 1 alpha 1 chain (*Col1a1;* Hs00164004_m1), *Col4a1* (Hs00266237_m1), elastin (*ELN*; Hs00899658_m1), microfibril-associated glycoprotein-1 (*MAGP-1*, Hs01027737_m1), *IL-1β* (Hs01555410_m1), *IL-6* (Hs00174131_m1), *IL-8* (Hs00174103_m1), *11β*-hydroxysteroid dehydrogenase type *(11β-HSD1;* (Hs00194153_m1), and human glyceraldehyde 3-phosphate dehydrogenase (*GAPDH*; Hs02786624_g1) endogenous control (Thermo Fisher Scientific). 

### 2.4. Conditioned-Media Experiment

HaCaT cells were seeded at 2 × 10^5^ cells/well in six-well plates and cultured for one day. Subsequently, the medium was replaced with serum-free DMEM supplemented with 1 μg/mL lipopolysaccharide (LPS) and active materials for 24 h. The cell suspension was centrifuged at 1300× *g* for 5 min, and the supernatant was collected for further analysis on NHDFs. The NHDFs seeded at 3 × 10^5^ cells/well before one day were treated with the conditioned media (CM) for 24 h and harvested. This method was adapted from the experimental protocols described by Oh, S. et al. (2023) and Seok, J.K. et al. (2015) [29,30].

### 2.5. Reconstructed Three-Dimensional (3D) Skin Experiment

The premade reconstructed 3D human skin model Neoderm^®^-ED was purchased from Tego Science (Seoul, Republic of Korea). The 3D skin was maintained and cultured following the manufacturer’s instructions. For the experiment, the test formulation containing Fuligo^®^ (2%), Ronacare Balmance^®^ (0.3%), hyaluronic acid (0.05%), and *Polygonum cuspidatum* root extract (PCE; 0.25%) was topically applied. For the control group, the same formulation without the active ingredients mentioned above was applied. The 3D skin was cultured for two days and stained with Masson’s trichrome. The stained sections of the 3D human skin model were imaged using the EVOS™ FL Auto2 Imaging System (Thermo Fisher Scientific). Dermal collagen area and epidermal thickness were qualified using the Image J Software version 1.54 (NIH, Bethesda, MD, USA).

### 2.6. Human Clinical Trial

This study was ethically approved by the LG H&H Institutional Review Board (LGHH20221110-AA-01-01; 10 November 2022). Nineteen healthy Korean women between the ages of 40 and 62 (average age: 49.2 years) were recruited. Firstly, we excluded volunteers who did not have enough fine wrinkles or enlarged pores according to our standards. The possible side effects of the study were thoroughly explained to each participant, and written informed consent was obtained before they participated in the clinical trial. Pregnant women or individuals undergoing dermatological procedures were excluded from the test. A double-blind, half-face design was used for the experiment. The test formulation containing specific ingredients, including Fuligo^®^ (2%), Ronacare Balmance^®^ (0.3%), hyaluronic acid (0.05%), and PCE (0.25%), was topically applied to the right side of the participants’ faces twice daily for four weeks. For the control, 0.1% retinol formulation without the active ingredients mentioned above was applied on the left side of the face. Before facial wrinkle measurements, all participants were required to rest for a minimum of 20 min in a controlled environment with a specific humidity (45 ± 5%) and temperature (22 ± 2 °C) after their faces were cleansed. Wrinkles on the participants’ faces were measured using an Antera 3D camera (Miravex, Dublin, Ireland). To analyze the effectiveness of the formulation, texture Ra and pore mean area parameters were used to assess changes in fine wrinkles under the eyes and the average pore area of the cheek, respectively. 

### 2.7. Statistical Analysis

The data are presented as the mean value and standard deviation derived from at least three independent experiments. Statistical analysis of data was performed using the Student’s *t*-test. A *p*-value less than 0.05 (* *p* < 0.05, ** *p* < 0.01, *** *p* < 0.001) indicated a significant difference.

## 3. Results and Discussion

### 3.1. Functional Study of Wrinkle-Related Gene SGPP2 in HaCaT Cells

*SGPP2* is a gene primarily expressed under inflammatory conditions [27]. While SGPP1 has been implicated in skin keratinocyte differentiation, the role of SGPP2 in this context remains unclear. However, SGPP2 has been shown to contribute to the production of IL-1β and IL-8, inflammatory cytokines induced by the TNF-α inflammatory cytokine in endothelial cells, and plays a major role in pro-inflammatory signaling. SGPP2 is reportedly expressed within 24 h, particularly within a short duration, in response to inflammatory stimuli such as LPS or TNF-α, and siRNA-mediated inhibition of *SGPP2* expression also suppresses the IL-1β and IL-8 inflammatory response. Since these inflammatory responses can be involved in various processes of wrinkle formation and pore size regulation, it is necessary to confirm whether *SGPP2* is also involved in the inflammatory response in skin cells to determine its association with skin wrinkle formation.

**Figure 1:**
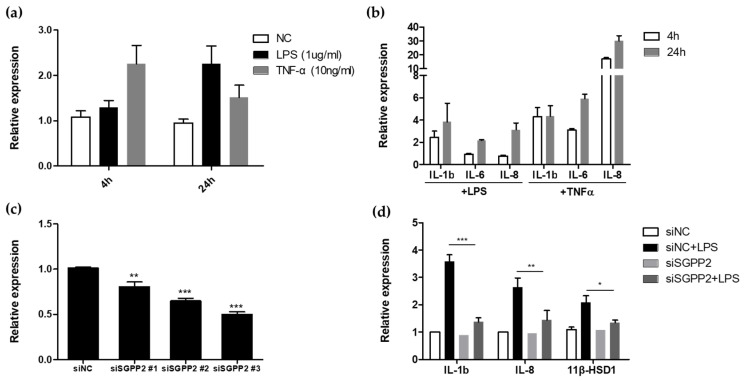
*SGPP2* expression and *SGPP2* knockdown effects in HaCaTs under inflammatory conditions. (**a**) Changes in sphingosine-1-phosphate phosphatase 2 (*SGPP2)* gene expression over time in lipopolysaccharide (LPS)-treated (1 ug/mL) and tumor necrosis factor (TNF)-α treated (10 ng/mL) HaCaT cells (**b**) Expression of other inflammatory cytokines in LPS-treated and TNF-α treated HaCaT cells (**c**) Downregulation of *SGPP2* expression using three siRNAs (si*SGPP2*) compared to negative control siRNA (siNC) (**d**) Expression of genes associated with skin inflammation and barrier stress-related genes in *SGPP2*-knockdown cells. Bars indicate standard deviation (n = 3). *** *p* < 0.001, ** *p* < 0.01, * *p* < 0.05; Student’s *t*-test.

Therefore, to investigate whether this phenomenon also occurs in keratinocytes, we examined the transcriptional level of *SGPP2* in keratinocytes treated with LPS and TNF-α at 4 and 24 h post-treatment (Figure 1a). Similar to the observations in endothelial cells [27], we found that *SGPP2* upregulation was more rapid upon TNF-α treatment. The transcriptional level of *SGPP2* was 2.25-fold higher 4 h after TNF-α treatment, while a similar transcriptional level was observed 24 h after LPS treatment. We also examined the transcriptional levels of *IL-1β*, *IL-6*, and *IL-8*, which are the cytokines that share downstream signaling pathways at the same time points. This analysis revealed a pattern of increased expression of inflammatory cytokines along with *SGPP2* upregulation (Figure 1b). The differences in the response patterns of *SGPP2* upregulation and inflammatory cytokine expression to LPS and TNF-α, respectively, are believed to be owing to differences in the response rates of NF-κB signaling induced by their respective receptors [31]. Based on the findings presented in Figure 1a,b, we confirmed that *SGPP2* upregulation and the expression of other inflammatory cytokines occur in skin keratinocytes upon LPS or TNF-α treatment.

To further investigate whether *SGPP2* downregulation directly regulates the inflammatory cytokines *IL-1β* and *IL-8*, we performed additional experiments using si*SGPP2*-transfected keratinocytes to assess their response to LPS. As shown in Figure 1c, si*SGPP2* transfection significantly reduced *SGPP2* mRNA expression compared with that in the control cells. Notably, *SGPP2* knockdown significantly attenuated LPS-induced expression of *IL-1β* and *IL-8* and inhibited *11β-HSD1* upregulation (Figure 1d), which is involved in the inflammatory response, interacting with NF-kB, regulating IL-6 production upstream, and is associated with skin barrier stress formation [32,33,34]. While the extent to which the enzymatic activity of SGPP2 influences S1P or sphingosine levels remains unclear, its minimal impact on skin and blood sphingosine/ceramide concentrations in knockout mice, along with previous studies highlighting the primary role of SGPP1 in S1P regulation, suggests SGPP2 functions more directly in downstream inflammatory signaling pathways [27]. These findings provide evidence that *SGPP2* contributes to LPS-mediated inflammatory cytokine production and skin barrier stress in keratinocytes.

### 3.2. Inflammation Exacerbates Skin Fibroblast-Mediated Wrinkle Formation

Skin wrinkles, a hallmark of aging, are attributed to the loss of dermal ECM, such as collagen and elastin [35]. Fibroblasts in the dermis are the primary producers of these fibers, and their function can be affected by various factors, including epidermal inflammation [36,37].

Skin fibroblasts exhibiting an “aged” phenotype, characterized by altered morphology, reduced proliferation activity, and high beta-galactosidase (β-gal) activity, have been studied using various methods, including continuous UV exposure, prolonged culture or isolation from elderly donors [38,39,40,41,42]. In vitro, prolonged culture induces a senescence-like state in skin fibroblasts, characterized by increased expression of calponin 1 (CNN1) and alpha-smooth muscle actin (α-SMA), associated with a reticular fibroblast phenotype and decreased expression of podoplanin (PDPN), associated with a papillary fibroblast phenotype. This suggests that in vitro prolonged culture-induced senescence-like skin fibroblasts may also contribute to aging-associated structural changes [41]. In this study, we used NHDFs that had undergone extensive passaging more than 20 times, resulting in a senescence-like state characterized by larger morphology, high expression of CNN1 and α-SMA, and low expression of PDPN (Figure S1).

**Figure 2:**
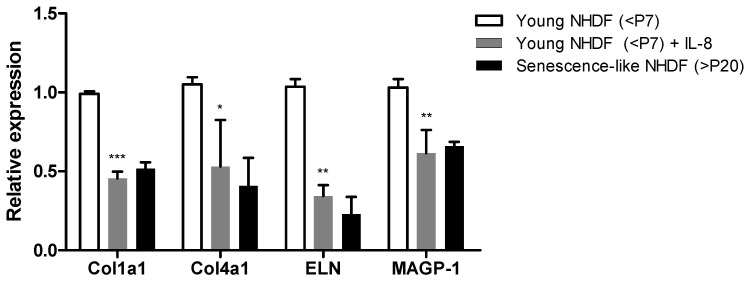
Expression of genes associated with human skin ECM under inflammatory conditions compared to senescence-like normal human dermal fibroblasts (NHDFs). Relative expression of *Col1a1*, *Col4a1*, *ELN*, and *MAGP-1* in young NHDFs with or without IL-8 (10 ng/mL) for 24 h and in senescence-like NHDFs, which were cultured for more than 20 passages. Bars indicate standard deviation (n = 3). Data were compared between young NHDFs treated with or without IL-8. *** *p* < 0.001, ** *p* < 0.01, * *p* < 0.05; Student’s *t*-test.

To elucidate how epidermal inflammation contributes to wrinkle formation through fibroblast dysfunction, we investigated how IL-8, an inflammatory mediator produced under the control of SGPP2 in the epidermis, affects the expression of ECM-related genes (*Col1a1*, *Col4a1*, and *ELN*) and *MAGP-1*, a crucial component in elastic fiber involved in pore size regulation, in fibroblasts [14,43]. We compared the expression of ECM-related genes in IL-8-treated fibroblasts with early passage (negative control) and senescence-like NHDFs (>P20). In senescence-like NHDFs, *Col1a1, Col4a1, ELN*, and *MAGP-1* were all downregulated, similar to that in early passage fibroblasts treated with IL-8 (Figure 2). While *Col1a1*, *Col4a1*, and *ELN* are known to be regulated by inflammation, the regulation of *MAGP-1*, an ECM-related gene that organizes elastic fibers and is essential for supporting pore structures [43] by IL-8 treatment, is novel. This experiment confirms that *Col1a1*, *Col4a1*, and *ELN* in skin fibroblasts, which are essential for wrinkle formation, and *MAGP-1*, related to sagging pores, are upregulated by the SGPP2-regulated inflammatory substance IL-8, thereby promoting an elderly-like phenotype in skin fibroblasts.

### 3.3. Screening Active Materials That Regulate SGPP2 Expression

We observed an upregulation of *SGPP2* and pro-inflammatory cytokine expression in keratinocytes under an LPS-induced inflammatory condition. This phenomenon downregulates ECM factors in fibroblasts, potentially contributing to skin wrinkle formation.

**Figure 3:**
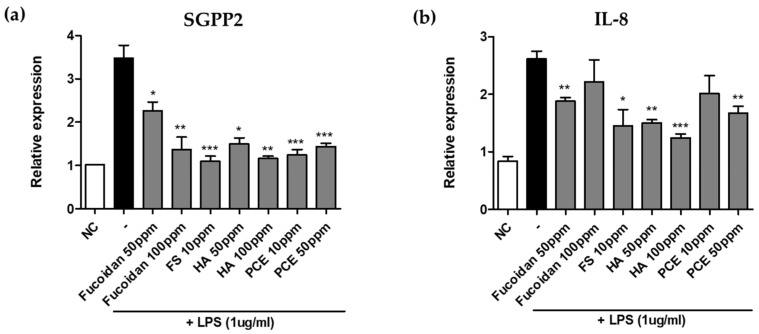
Screening materials with the ability to downregulate *SGPP2* expression and *IL-8* inflammatory cytokine. (**a**) Relative expression of *SGPP2* in keratinocytes (HaCaT) treated with Fucoidan, Feruloyl Serotonin (FS), Hyaluronic acid (HA), and *Polygonum cuspidatum* root extract (PCE) at the mentioned concentrations. All samples except Negative control (NC) are treated with LPS (1 ug/mL). (**b**) Anti-inflammatory effect of *SGPP2*-downregulating materials that decreases *IL-8* expression in LPS-treated keratinocytes. Bars indicate standard deviation (n = 3). *** *p* < 0.001, ** *p* < 0.01, * *p* < 0.05; Student’s *t*-test.

To investigate the modulation of *SGPP2* expression and its impact on skin wrinkle formation and pore sagging, we evaluated the effects of various anti-inflammatory substances on *SGPP2* regulation in LPS-induced inflammation. We assessed the ability of fucoidan, feruloyl serotonin, hyaluronic acid, and PCE to downregulate *SGPP2* expression in LPS-treated keratinocytes. These substances are known for their anti-inflammatory properties, including reducing LPS-induced pro-inflammatory cytokine expression (fucoidan and feruloyl serotonin) and inhibiting LPS-toll-like receptor 4 binding (hyaluronic acid) [44,45,46]. Our findings revealed that fucoidan (50 and 100 μg/mL) significantly decreased *SGPP2* expression by 35% and 61%, respectively, compared with LPS alone (Figure 3a). Similarly, feruloyl serotonin (10 ppm) downregulated *SGPP2* expression by 69%. Hyaluronic acid (50 and 100 ppm) also effectively reduced *SGPP2* levels by 58% and 67%, respectively. Additionally, PCE (≥10 ppm) exhibited a significant decrease in *SGPP2* expression by 65%. We also found that these materials also inhibited LPS-induced IL-8 expression in keratinocytes (Figure 3b). Further analysis of other anti-inflammatory and wrinkle-improving agents demonstrated that L-hydroxyproline, retinol, cedrol, asiaticoside, and ginsenoside Rh1 also significantly downregulated *SGPP2* expression (Figure S2). 

### 3.4. Antiaging Effect of SGPP2 Expression-Regulating Materials In Vitro

#### 3.4.1. Recovery of Inflammation-Induced Decrease in ECM-Related Gene in NHDFs 

Paracrine signaling, a form of cell-to-cell communication, is crucial in various biological processes. In skin aging and wrinkle formation, the paracrine effects of keratinocytes on fibroblasts are particularly significant. CM experiments are used in vitro to investigate paracrine signaling between two cell populations [29]. Skin fibroblasts play a central role in regulating elastic fibers and are key components associated with wrinkle formation [47]. Our previous findings in Figure 2 demonstrated that IL-8, an inflammatory cytokine potentially induced by inflammation in keratinocytes, downregulates elastic fibers in fibroblasts.

To exclude the direct effects of these substances on collagen regulation in fibroblasts and investigate the paracrine regulation mediated by the inflammation-modulating effect of keratinocytes, we examined whether these substances could mitigate the LPS-induced downregulation of elastic fibers in fibroblasts through paracrine signaling.

**Figure 4:**
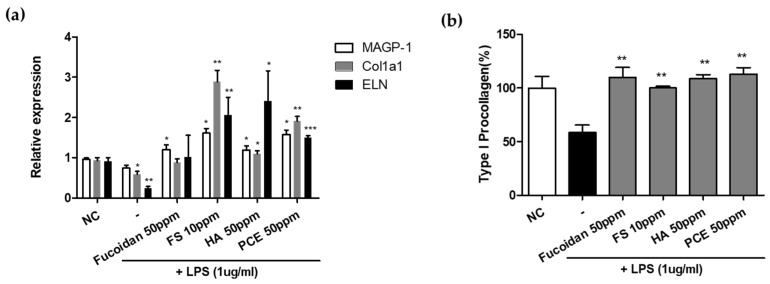
Analysis of ECM-enhancing effects in dermal fibroblast of *SGPP2* expression-regulating materials through in vitro experiments. (**a**) Relative expression of *MAGP-1*, *Col1a1,* and *ELN* genes in normal human dermal fibroblast treated with conditioned medium (CM) for 24 h produced by LPS-treated HaCaT cells cultured in Fucoidan, Feruloyl Serotonin (FS), Hyaluronic acid (HA) and *Polygonum cuspidatum* Root extract (PCE) for 24 h at the mentioned concentrations. (**b**) Analysis of changes in type I collagen synthesis in human dermal fibroblasts treated with CM. Bars indicate standard deviation (n = 3). Data were compared between LPS-treated groups treated with and without each substance. *** *p* < 0.001, ** *p* < 0.01, * *p* < 0.05; Student’s *t*-test.

CM from LPS-treated keratinocytes significantly downregulated *MAGP-1*, *Col1a1*, and *ELN* expression in fibroblasts by 23%, 37%, and 75%, respectively, compared with the control CM (Figure 4a). Fucoidan-containing CM markedly reversed the LPS-induced decrease in *MAGP-1* expression. Additionally, CM containing feruloyl serotonin, hyaluronic acid, or PCE substantially significantly increased the expression of *MAGP-1*, *Col1a1*, and *ELN*, which were downregulated by LPS treatment. 

These findings suggest that modulating substances that regulate *SGPP2* in keratinocytes can paracrinely influence the regulation of wrinkle-related elastic fibers in dermal fibroblasts. This restoration of elastic fiber expression by modulating substances in CM is possibly attributed to the reduced secretion of inflammatory cytokines such as IL-8 and IL-1b due to *SGPP2* inhibition and downregulation of inflammatory signaling in LPS-treated keratinocytes.

#### 3.4.2. Effect of Enhanced Type I Procollagen Synthesis 

We discovered that *SGPP2*-regulating substances applied to keratinocytes in the epidermis can paracrinely affect the regulation of wrinkle-related elastic fibers in dermal fibroblasts. 

To validate whether the observed modulation of elastic fiber expression in CM also translates to protein synthesis at the protein level, we assessed type I collagen protein levels, the primary subtype in human skin, using an enzyme-linked immunosorbent assay.

Keratinocytes were treated with LPS alone or LPS in combination with modulating substances (fucoidan, feruloyl serotonin, hyaluronic acid, or PCE) to generate the CM. This CM was applied to dermal skin fibroblasts. Treatment with 50 μg/mL fucoidan, 50 μg/mL feruloyl serotonin, 50 μg/mL hyaluronic acid, and 50 μg/mL PCE resulted in 1.23-fold, 1-fold, 1.21-fold, and 1.31-fold increases in type I collagen restoration compared with that of LPS-only CM (Figure 4b). These results demonstrate that the paracrine effects of keratinocytes treated with these four modulating substances regulate collagen transcription and impact protein expression levels in fibroblasts.

### 3.5. Dermal Collagen Enhances the Efficacy of the Selected Materials within 3D Skin Model

While 2D monolayer cell culture systems offer convenience for studying molecular mechanisms, they may not accurately reflect in vivo conditions. Therefore, to assess the efficacy of our material in a more realistic environment, we used a reconstructed 3D human skin model (Neoderm^®^-ED) [48,49]. 

**Figure 5:**
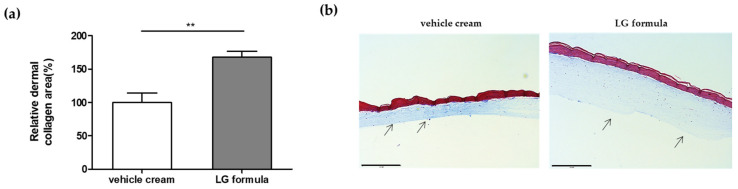
Dermal collagen upregulation in 3D skin model by treatment with formulations containing *SGPP2* modulating materials. (**a**) Effect of two cram formulations on increasing dermal collagen area in 3D skin equivalent; (**b**) Representative images cross sections of 3D skin equivalent; scale bar = 275 um. Black arrows indicate the dermis as visualized with Masson Trichrome stain. Bars indicate standard deviation (n = 3). ** *p* < 0.01; Student’s *t*-test.

We applied creams containing our four active materials (LG formula) to the 3D skin model and compared the results to those obtained with a vehicle cream. Through a comprehensive evaluation of in vitro efficacy concentrations, stability, and solubility, an optimal combination of the four active materials was determined and formulated. After treatment for 48 h, the relative area of dermal collagen significantly increased by +68.1% in the LG formula-treated samples (Figure 5a). This enhancement in the dermal collagen area was further confirmed through histological analysis (Figure 5b).

These results demonstrate that the four active materials, when incorporated into a cosmetic formulation, can promote collagen production and potentially contribute to wrinkle improvement in a 2D cell culture and also in a more human-like 3D culture environment.

### 3.6. Improvement in Skin Wrinkles and Pores Using LG Formula-Containing Materials That Modulated SGPP2 Expression

Subsequently, we conducted an experiment to determine if the formulation containing four types of active materials improved the appearance of facial wrinkles and pores. Based on the 3D human skin model test, we formulated LG formulas containing each of the four active materials and tested them to determine if they improved the appearance of wrinkles on the under eye and pores. To accurately measure the efficacy of the LG formula, a half-test method experiment was conducted, where 0.1% retinol cream of the same base formula was applied to the left side of the face, and the LG formula cream containing four active substances was applied to the right side of the face. To assess the efficacy of four active material complexes, we selected 0.1% retinol cream as a control, given its established reputation as a highly effective anti-wrinkle ingredient. The changes in the area of facial wrinkles and pores were measured with Antera 3D, among which the Ra value of texture, which is a common parameter for measuring fine wrinkles [50], and the average pore area, which is a suitable parameter for showing stretched pores or pore elongation, were measured [15,51]. 

**Figure 6:**
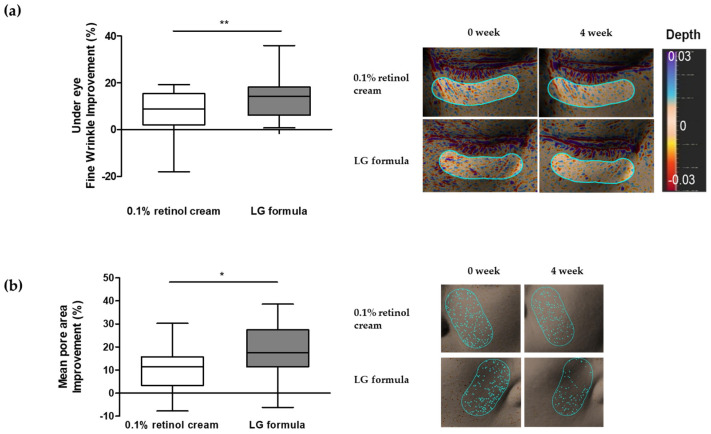
Facial wrinkle and pore improvement by the LG formula containing *SGPP2* expression-regulating materials. (**a**) Comparison of the undereye fine wrinkle improvement rate; (**b**) Comparison of the mean pore area improvement rate between LG formula- and 0.1% retinol-treated groups after four weeks. Representative images were acquired using an Antera 3D camera both pre- and post-treatment (4 weeks). Bars indicate standard deviation (n = 19). ** *p* < 0.01, * *p* < 0.05; Student’s *t*-test.

After applying both creams once daily for four weeks, the results showed a 14.3% improvement in fine wrinkles in the under-eye area (Figure 6a) and a 17.22% improvement in the mean pore area (Figure 6b). In contrast, the 0.1% retinol cream alone showed a relatively low improvement rate of 6.52% and 10.13% in the control group, showing significant differences in under-eye fine wrinkle and mean pore area improvements, respectively. 

Although retinol alone can improve under-eye fine wrinkles and stretched pores, individual differences in retinol reactivity exist, and the perception of wrinkle improvement may be lower than expected owing to irritation (such as dryness, redness, and hyperkeratinization) caused by retinol. However, this study shows that LG formula with various ingredient combinations can provide similar or better results than retinol, a well-known anti-wrinkle ingredient, in reducing under-eye fine and pore wrinkles without irritation. Although a significant level of improvement was confirmed after four weeks of use, the limited sample size and experimental duration of this study preclude definitive conclusions about the long-term efficacy of the product. While the observed improvements are encouraging, further research with a larger sample size and extended treatment periods is necessary to fully assess the potential benefits of the formula [52,53]. 

In this study, based on the hypothesis that *SGPP2* is involved in the development of facial wrinkles, we showed how four materials that modulate *SGPP2* can ameliorate facial wrinkles in vitro and in vivo. However, the effect of *SGPP2* as a phosphatase enzyme and the limited conditions, such as sample size and the duration of clinical study, are the limitations of this study. An in-depth investigation of *SGPP2* signaling and S1P after inflammatory stimuli is required to further clarify the role of *SGPP2* in wrinkle formation.

## 4. Conclusions

In this study, we highlighted the potential of regulating *SGPP2* in keratinocytes, which influences LPS-induced pro-inflammatory signaling and inflammatory cytokines such as IL-8, commonly known as SASP. This pro-inflammatory cytokine acts on dermal fibroblasts, prompting the expression of ECM-related genes associated with an aged phenotype.

We identified four active substances that effectively reduce *SGPP2* expression under inflammatory conditions. Using CM experiments, we confirmed their potential to alleviate the inhibition of dermal collagen production caused by epidermal inflammation. To assess the applicability beyond 2D cultures, the application of a cosmetic formulation (LG formula) containing the four selected substances demonstrated a significant improvement in collagen synthesis in a 3D skin model. This indicates their effectiveness in an environment mimicking human skin conditions.

Finally, a clinical evaluation was conducted using the LG formula. Compared with 0.1% retinol, a well-established anti-wrinkle treatment, the LG formula demonstrated superior efficacy in improving fine wrinkles under the eyes and enlarged/elongated pores.

This study elucidates the potential of *SGPP2* modulation as a novel anti-wrinkle strategy that mitigates inflammation. The identification of these four active ingredients is promising for the development of effective antiaging products that address a broader spectrum of facial wrinkles, including pore enlargement and elongation.

## Data Availability

The data that support the findings of this study will be made available by the corresponding author upon request.

## Supplementary Materials

The following supporting information can be downloaded at https://www.mdpi.com/article/10.3390/cimb46080539/s1, Figure S1. Changes in morphology and gene expression (CNN1, αSMA, PDPN) of NHDFs under prolonged culture conditions (more than 20 passages). Figure S2: The effect of anti-inflammatory and wrinkle-improving materials on *SGPP2* expression.
