# Supplementary material for: Efficacy of SGPP2 Modulation-Mediated Materials in Ameliorating Facial Wrinkles and Pore Sagging
Source: Curr Issues Mol Biol. 2024 Aug 20;46(8):9122–35. doi: 10.3390/cimb46080539 (PMC11352500; doi:10.3390/cimb46080539)
Supplement: Supplementary file 1 [file cimb-46-00539-s001.zip › cimb-3159449-supplementary.pdf]

current issues in molecular biology

MDPI

Supplementary Material: Efficacy of SGPP2 Modulation-mediated Materials in Ameliorating Facial Wrinkles and Pore Sagging Title

Juhyun Kim, Sanghyun Ye, Seung-Hyun Jun* and Nae-Gyu Kang*

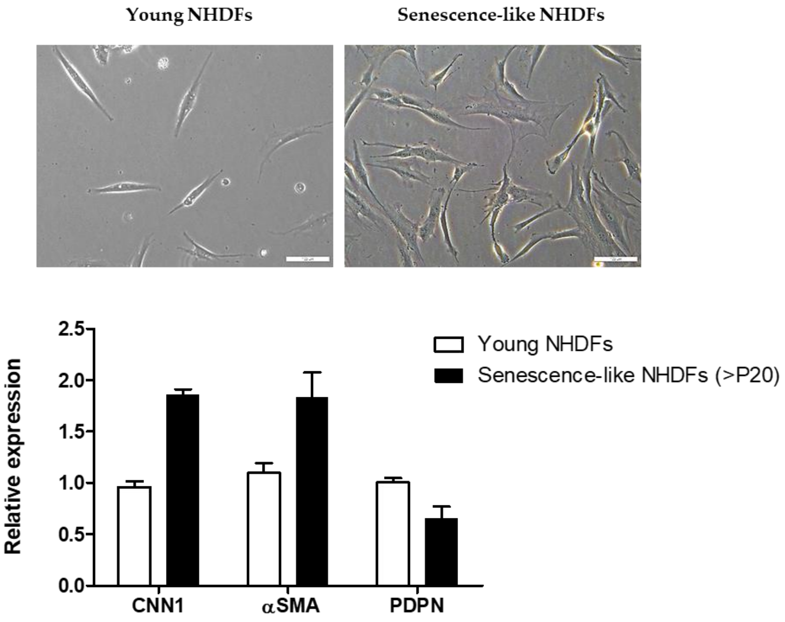

Figure S1. Changes in morphology and gene expression (CNN1, αSMA, PDPN) of NHDFs under prolonged culture conditions (more than 20 passages) Representative microscopic images (up) and relative expression of CNN1, αSMA and PDPN in young NHDFs and senescence-like NHDFs**.**

Curr. Issues Mol. Biol. **2024**, *46*, 9122–9135. https://doi.org/10.3390/cimb46080539 www.mdpi.com/journal/cimb

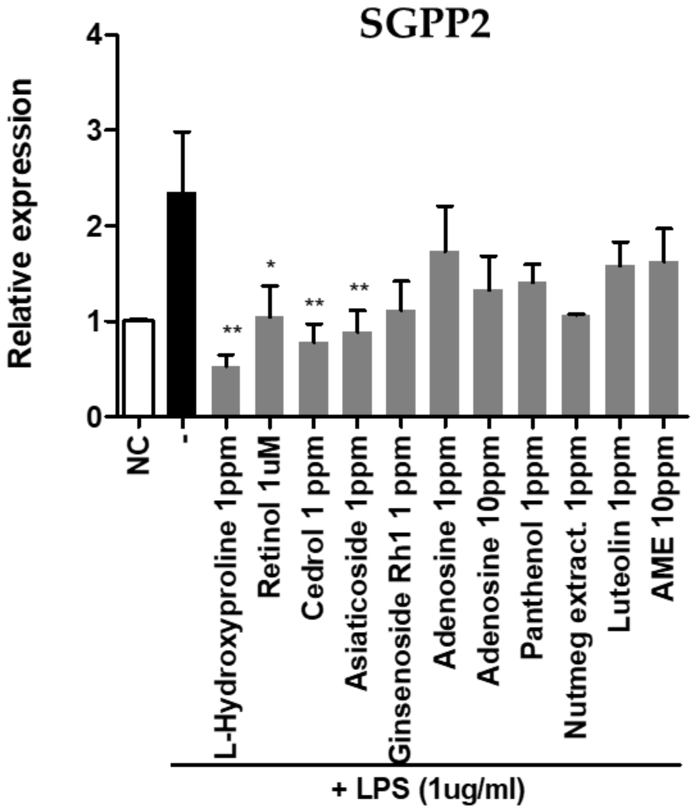

Figure S2. The effect of anti-inflammatory and wrinkle-improving materials on *SGPP2* expression. Relative expression of *SGPP2* in keratinocytes (HaCaT) treated with L-Hydroxyproline, Retinol, Cedrol, Asiaticoside, Ginsenoside Rh1, Adenosine, Panthenol, Nutmeg extract (*Myristica fragrans* extract), Luteolin and *Astragalus membranaceus* root extract (AME). All samples except Negative control (NC) are treated with LPS (1ug/ml). Bars indicate standard deviation. ** $p < 0.01$, * $p < 0.05$; Student's t-test
